# Influences of Cultural Factors on Vaccination Practices Among Parents Prior to School Age: A Systematic Review and Meta-Analysis

**DOI:** 10.7759/cureus.75845

**Published:** 2024-12-17

**Authors:** Haia Mahdi Hindi Albalawi, Khulud Ahmad Rezq

**Affiliations:** 1 Faculty of Nursing, University of Tabuk, Tabuk, SAU

**Keywords:** cultural factors, parents, saudi arabia, school age, vaccine

## Abstract

Understanding the cultural influences on parents in Saudi Arabia regarding adherence to childhood vaccination programs is crucial for the health and safety of the whole society. This study aims to explore the impact of cultural factors on parents' attitudes toward vaccinating children before school age. A systematic review and meta-analysis were conducted on cross-sectional and interventional studies. Electronic databases were comprehensively searched using specific keywords after obtaining PROSPERO Registration ID: CRD42024517434. The inclusion criteria focused on studies that examined cultural factors influencing childhood vaccination in Saudi Arabia. Across 30 articles, we identified seven key themes: economic and political aspects, cultural values, the socio-cultural position of women, the impact of religious and philosophical factors, knowledge and education levels of parents, technological influences, and knowledge levels of health care providers. The pooled cultural factors were analyzed using random-effects meta-analysis, and we conducted meta-regression studies to explore the effects of relevant variables. The meta-analysis revealed significant associations between parental age, education level, and the number of children with vaccination compliance. Parents aged 30-40 or over 40, those with higher education levels, and those with more than four children were more likely to be compliant. Nevertheless, parental occupation did not exhibit a significant association with vaccination compliance. This study provides a comprehensive understanding of the intricate cultural factors influencing childhood vaccination in Saudi Arabia. The findings highlight the importance of tailoring public health strategies to address specific cultural nuances and promote vaccination adherence.

## Introduction and background

Worldwide, several vaccinations have been launched to reduce avoidable illnesses that have caused morbidity and death in various regions. Numerous factors influence the efficiency and adherence to regular childhood immunization regimens [1]. In low- and middle-income nations, low vaccination program adherence can be attributed to a range of logistical, biological, epidemiological, and economic issues [1]. Recent research, however, indicates that social and cultural factors also play a significant role in shaping vaccination decisions for children, even in nations of higher income levels. This research focuses on the cultural factors influencing parents in the Kingdom of Saudi Arabia (KSA) to delay vaccinating their children within the framework of cultural influences [1].

According to the World Health Organization's (WHO) 2019 data, Saudi Arabia boasts a 96% to 98% childhood immunization coverage rate. Although 2018 results showed a slight reduction compared to earlier years, the WHO-UNICEF calculated that the national coverage of the third dose of diphtheria, tetanus toxoids, and pertussis vaccine (DTP3) consistently remained above 95% from 2005 to 2018 [1]. To address vaccine-preventable illnesses, the WHO recommends a regular immunization schedule for children [2].In Saudi Arabia, the National Immunization Schedule mandates vaccinations at birth, 2, 4, 6, 9, 12, 18, and 24 months, as well as at age 7, when children are ready to begin school [2]. This systematic schedule ensures timely protection against vaccine-preventable diseases during critical stages of a child's development.

The Ministry of Health (MOH) in KSA requires parents to provide a vaccination certificate, certified by a medical professional, before enrolling their children in school. Despite this mandate, delays in childhood immunizations remain a concern, jeopardizing children's health when vaccines are not administered on time [1]. While nationwide research indicates high vaccination coverage, some parents oppose vaccinations, resulting in lower adherence in specific cases [3-7]. For example, 9% of respondents in one study reported that they would not encourage their relatives to vaccinate their children, and 16.1% opposed the child immunization program. Regarding adherence to the recommended schedule, 79.6% of participants ensured that children completed the program, while 20.4% did not [3-6]. Suboptimal compliance persists as a significant challenge for health authorities in KSA. One study revealed that only 40.9% of children received their vaccines on time or with minimal delays, while 59.1% experienced delays of up to a month [8]. These delays underscore a lack of adherence and present a critical challenge to achieving herd immunity, which relies on widespread, timely vaccination. Even with the mandatory vaccination policy improving overall coverage, delayed immunizations are considered missed opportunities, leaving children vulnerable to preventable diseases [9,10].

Vaccine hesitancy, defined as a delay in accepting or refusing vaccines, arises from various factors, including misinformation and sociocultural beliefs [11]. Numerous variables contribute to vaccination hesitancy, ranging from technical advancements to sociocultural influences [6,12]. According to research by Al-Saeed et al. (2018) [12], illness and misinformation led to vaccines being unavailable in clinics, causing delays in vaccinating children. For instance, 2.5% of parents deliberately postponed vaccinations because they believed the shots were unnecessary, while 6.5% cited false information and concerns that vaccines might harm their children's developing brains [12]. Based on these findings, it can be inferred that the MOH in KSA has not fully succeeded in reducing the prevalence of communicable diseases through immunization and in improving the welfare of children and the broader community. Consequently, investigating the cultural and societal factors contributing to this issue is essential [13]. The MOH has established guidelines aimed at eradicating vaccine-preventable childhood illnesses. While most school-age students meet these objectives, there is a growing gap in achieving vaccination goals among preschool-age children, including those from birth to seven years old. Understanding cultural nuances is therefore critical to identifying the Saudi cultural factors that contribute to parents delaying vaccinations.

The specific aims

The aims of this paper are: i) To examine cultural factors that impact parents’ perceptions towards vaccinating their children prior to school age on a routine schedule. ii) To identify various cultural factors that affect childhood vaccination adherence rates in the KSA.

Theoretical framework

The literature review that has been conducted for this paper focused specifically on culture. The term "culture" has been used widely in different disciplines, and it is employed in multiple meanings and uses. Leininger defined culture as "the learned, shared, and transmitted values, beliefs, norms, and lifeways of a particular culture that guide thinking, decisions, and actions in patterned ways" [14]. This paper is guided by Leininger's Culture Care Diversity and Universality Theory as its theoretical basis. Leininger thought trans-cultural nursing care may lead to therapeutic health and healing effects. Accordingly, the theory's objective is to investigate how cultural elements affect people's beliefs, behaviors, health, and sickness, and then to offer thoughtful treatment that is suitable for any culture and safe for its members [14]. Leininger's approach highlights culture as an all-encompassing notion that created the connection between nursing practice and knowledge [14]. Worldviews and social structures are components of culture, which are acquired through natural environments. These can include, among other things, social, legal, philosophical, familial, religious, and economic considerations [14]). Understanding Saudi culture and the attitudes and ideas of Saudi parents is crucial to removing the obstacles that cause delays in child immunizations. Leininger proposed three modes that support the upkeep of care that is culturally appropriate. These modes are illustrated in "(1) culture care preservation and/or maintenance, (2) culture care accommodation and/or negotiation, and (3) culture care restructuring and/or repatterning" [14]. These three approaches are crucial for establishing the professional judgments and activities that nurses must take in order to enable cross-culturally appropriate care. To improve health outcomes that are mutually agreed upon by caregivers and patients, these modes promote and facilitate culturally congruent care through adaptation and negotiation with others, aid in maintaining effective treatment, and alter or reorganize people's lifeways. Leininger's Sunrise Enabler-Model offers an anthropological foundation for evaluating the cultural facets of Saudi parents, nurses, and the Saudi community that might have contributed to preschoolers' tardy adherence to immunization schedules. The Sunrise Enabler's rationale is to guarantee a thorough comprehension of the elements that have led to the postponed child immunization schedule; thus, it provides a basis for developing treatment that is culturally sensitive [15].

## Review

Methods

**Figure 1:**
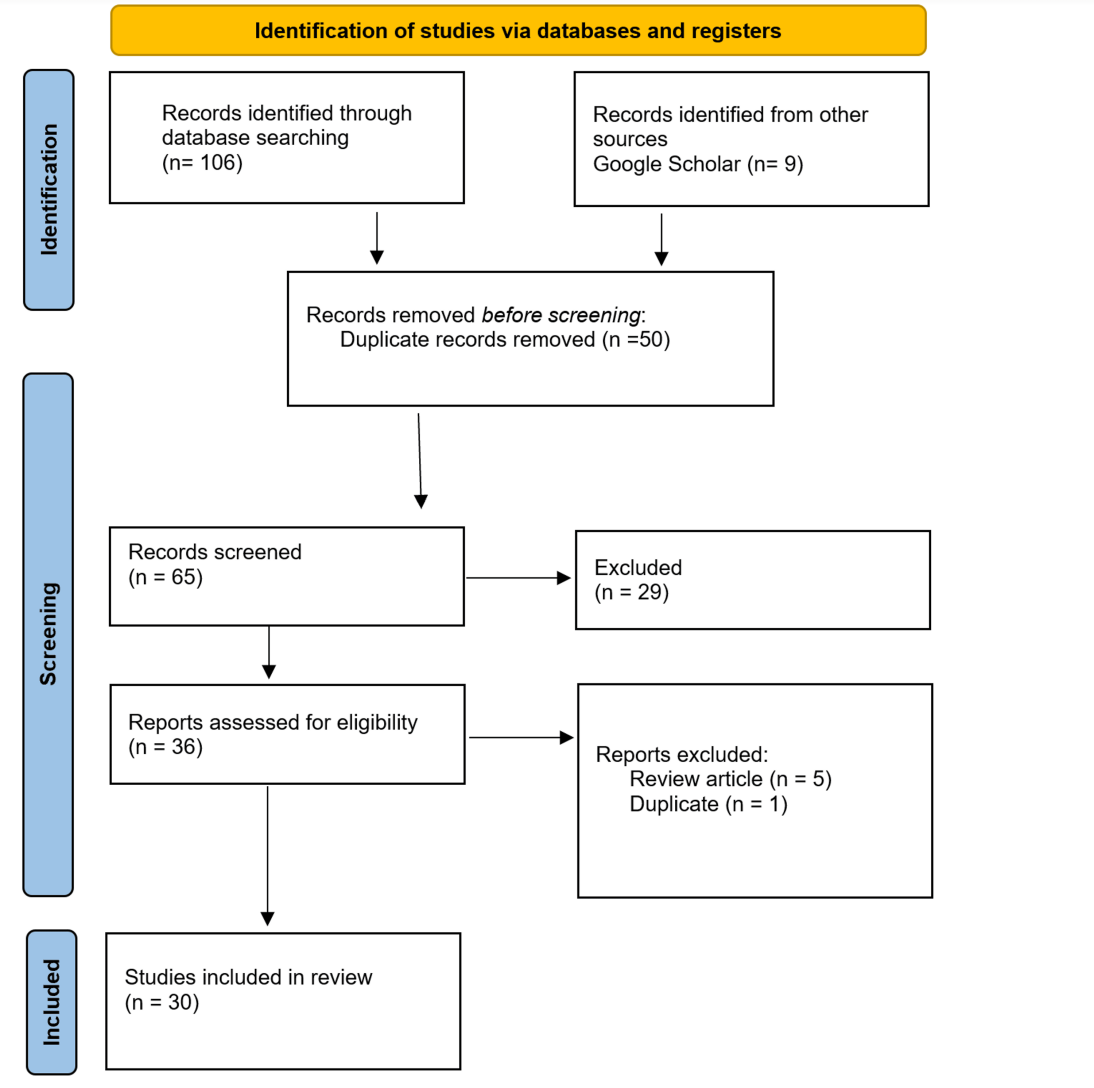
PRISMA flow diagram

The systematic review/meta-analysis search strategy was conducted by Haia Mahdi Hindi Albalawi and Khulud Ahmad Rezq. Independent searches were carried out by both writers on several databases, including Google Scholar, CINAHL, PubMed, EMBASE, ScienceDirect, Gale Academic One File, and Science Citation Index. The search was performed in August 2019, and vaccination, Saudi Arabia, culture, parents, views, Islam, hesitation, and kids were among the terms that were utilized. Peer-reviewed publications, English-language articles, and works containing at least one keyword in the title or abstract were all required for inclusion, in cases of disagreement between Haia Mahdi Hindi Albalawi and Khulud Ahmad Rezq. During the screening process, consensus was reached through thorough discussion. If disagreements persisted, a third party (Dr. Ahmed Taher), an expert in the field, was consulted to provide a final decision. The referee, (Dr. Ahmed Taher), was selected based on his expertise in the subject matter. Dr. Ahmed Taher was not involved in the initial search strategy but was consulted in cases where disagreements needed resolution. The search strategy followed the PRISMA guidelines (Figure 1), and after screening titles and abstracts, 29 papers were removed because they had no bearing on the review's objectives, while 50 studies were omitted for being duplicates. 30 publications that satisfied the inclusion criteria were found in the final search, offering a thorough overview of various study designs encompassing various KSA areas.

Quality assessment

We used the Newcastle-Ottawa Scale (NOS) to assess the quality of the included studies (16). It is based on three main domains: the first assesses the participant selection process, the second evaluates the comparability among study groups, and the last assesses the statistical analysis and the outcomes. The studies were judged to be of low quality if they scored <4 stars, medium quality if they had a score between 4 and 7 stars, and high quality if the score was >7 stars.

Statistical analysis

We used odds ratio (OR) and mean difference (MD) analysis for dichotomous and continuous data, respectively. In every instance, the confidence interval (CI) was 95%. Review Manager software was used for all data analysis. A fixed-effects model was used to examine homogeneous data, whereas a random-effects model was applied to assess heterogeneous data. To evaluate the heterogeneity, we employed the I2 index and Chi-square tests. I2 > 50% and p < 0.1 values were deemed heterogeneous. To address the varied results, we attempted Cochrane's leave-one-out technique.

Findings

In order to comprehend and record the themes that illustrated the effects of numerous cultural elements and their effect on childhood immunization in the KSA, an interpretative synthesis was created using Leininger's Sunrise Enabler Model (Table 1). The content gleaned from the 30 articles covered a broad range of cultural themes, including knowledge, religion, communication problems, traditional tendencies, family management structure, legality, and the roles of healthcare providers that were found to be associated with the degree of adherence to the recommended vaccination schedule for children. It is essential to comprehend this literature evaluation in its entirety in order to pinpoint any knowledge gaps regarding the causes of vaccine reluctance. In the Kingdom of Saudi Arabia, parent-related obstacles to children’s immunization are another goal of the research study. Seven themes were found to be present in all 30 articles.

**Table 1:** Mapping of cultural themes from the sunrise enabler model to literature review findings on childhood immunization in the Kingdom of Saudi Arabia

The Sunrise Enabler Model Themes	Literature Reviews Themes
Educational Factors	Knowledge Gaps Among Healthcare Providers. Knowledge and Education Level of Saudi Parents toward Vaccinating Preschool Children
Economic factors Political &legal factors	Saudi Arabian Economic and Political Aspects
Cultural values, beliefs & lifeways	Saudi Cultural Values, Beliefs, Traditions and Norms
Kinship & social factors	The Socio-Cultural Position of Women in Family and Society
Religious & philosophical factors	The Impact of Religious and Philosophical Factors on Saudi Arabia’s Health Practices
Technological factors	Technological Factors

Saudi Arabian economic and political aspects

Many nations have established routine vaccination regimens in accordance with WHO standards and in response to issues related to regional diseases. In Saudi Arabia, the National Immunization Schedule was implemented for children from the time of birth until they reached school age. All individuals received free vaccinations from the government [2]. This suggests that the affordability problem is unaddressed. Additionally, mobile clinics are employed to provide preventative healthcare in some isolated places. Preschool vaccine adherence is quite high in Saudi Arabia, as it is in wealthy nations [1,5,14]. The legal need for a kid to receive a vaccine before beginning school is the cause of the high adherence rate [4]. Cases of delayed infant vaccination were documented in spite of the government-imposed restrictions. These cases might be attributed to several variables, such as social and cultural features [6-7,16]. Research that was carried out in Tabuk, which is in the KSA's northwest, sought to identify and analyze ailments that affect youngsters. According to Al Tabbal and Al Humedi (2017) [17], researchers emphasized the significance of carrying out more studies to address the impact on the health system when access to healthcare in rural regions remains an issue. As indicated by the delays, this policy has not solved the issue of timely immunization from birth to seven years of age despite the legal necessity that resulted in the high vaccination coverage [12].

Saudi cultural values, beliefs, traditions, and norms

Cultural conventions, religious convictions, traditions, and values all have a big impact on how people seek health care. Saudis seeking medical attention or routine vaccinations are not discouraged from doing so by their Islamic beliefs, according to the Ministry of Health (MOH) of the Kingdom of Saudi Arabia (1998) [18,19]. It has been emphasized that other cultural factors, rather than the Islamic faith, affect preschoolers' delayed immunization schedules in the Kingdom of Saudi Arabia. Take the function of both conventional and spiritual healing, for example. Even with the expansion of modern healthcare facilities in the Kingdom of Saudi Arabia, some individuals still place a high value on herbalists, who occasionally have the authority to order the cessation of hospital care [20-22]. This situation is further compounded by the Saudi family structure and the status of women in terms of decision-making. Cultural dispositions of women to believe in or receive instructions from others, such as spouses, may contribute to this phenomenon in topics pertaining to health rights and empowerment [20,22]. These sociocultural factors highlight how important health education is for empowering women. Some parents refuse vaccinations due to cultural beliefs and belief systems; instead, they seek advice from traditional healers and wait for orders from dependable family members.

The socio-cultural position of women in family and society

Making decisions concerning one's health is greatly influenced by the role that family members play. In Saudi culture, family members have a big role in the way patients' care is managed. Some women assign important health choices to men because they think they know better, for example, their husbands or dads [20]. On the other hand, women are more likely than dads to accompany their children to receive vaccinations, according to a study done in Taif, which is in the western part of the Kingdom of Saudi Arabia [20]. In the majority of Tabuk's medical facilities, staff members only let moms inside the vaccination department, where women make up most of the medical staff, and forbid dads from entering [20]. Saudi Arabian society is marked by significant gender segregation across various settings, including government agencies, entertainment venues, and educational institutions [21-22]. Social standards have several negative effects on the health of Saudi women and children and may discourage them from visiting medical facilities for treatment. These explanations are typically linked to the idea that male guardians are the ones who make decisions about their health. Furthermore, some orthodox households only let women go to and from work or medical facilities when accompanied by male guardians [23]. These social norms are significant, and they mislead women on their important health visits and for their children's health. Such practices imply that males who are the key decision-makers are not sensitive to the importance of vaccines. However, tiny cities with a predominantly tribal population are primarily affected by the cultural trend where only women are allowed to work in vaccination offices. It is important to note that the Ministry of Health (MOH) has not issued a policy directive on this matter. Rather, cultural attitudes about how men and women should interact have their roots in tribal traditions and conventions rather than in Islamic doctrine [20,22,23-25]. In Saudi Arabia, women were not permitted to drive for a very long period and had to go to certain locations with a male guardian. It has been shown that these restrictions serve as societal obstacles that keep women confined to their homes and prevent them from taking an active role in issues pertaining to their own health. Even though women were now permitted to drive in 2018 due to changes in the legislation, certain families may continue to uphold outdated customs, beliefs, and social mores that prohibit women from traveling around society without a male guardian. Moreover, [22] pointed out that sociocultural customs that restrict women's freedom of movement jeopardize their capacity for physical activity and their capacity to attend doctor's visits. Furthermore, it's possible that a lack of resources, such as social support, motivation, and public transportation, contributed to the delay in getting kids to their scheduled immunizations. Some academics have referred to the restrictions as lifestyle-related issues that negatively impact women's and children's health [23]. These approaches advance healthcare in accordance with God's will, improving outcomes in terms of patient acceptance of medical procedures.

The impact of religious and philosophical factors on Saudi Arabia’s health practices

Religious, social, and philosophical elements have been identified as significant global determinants of health adherence patterns. It has been discovered that religious convictions influence vaccination rates in some low-income nations. Research from the Middle East showed that Muslim children had worse health and a higher death rate than Christian children [26,27]. That may be the case for some Middle Eastern nations, but it is impossible to make a broad generalization from this, and qualitative research is required to determine whether parents' views are anti-vaccination or have religious overtones. According to Alfahl and Alharbi (2017) [28], parental religious background may have an impact on a child's normal immunization schedule in the Kingdom of Saudi Arabia. According to data, for example, religion has a 2% influence on child vaccination rates in the Kingdom of Saudi Arabia [11]. Saudi parents who believe in predestination are not discouraged from receiving vaccinations or any other preventative care, according to the Ministry of Health [2]. The laws now in effect in the Kingdom of Saudi Arabia uphold people's rights and progress in areas pertaining to health, work, freedom, and travel in a way that is consistent with Islamic religious teachings and cultural values. The foundation of these laws is Saudi Vision 2030, which aims to enable the Saudi people's personal and collective rights [29]. On the other hand, certain leaders' ignorance and misunderstandings regarding health rights and their opposing views of Islam provide the wrong picture of the religion's actual teaching [23]. Thus, other national and traditional difficulties, rather than only the Islamic faith, are related to the cultural and social variables that cause delays. In many nations, the theological debates surrounding the administration of certain vaccinations have become major problems. For example, vaccinations containing porcine gelatin have been met with opposition by the populace in Islamic-majority nations like Afghanistan and Pakistan [30]. This is because porcine gelatin comes from pigs, and eating anything derived from pork is forbidden in Islam. Scholars have pointed out that these vaccinations are allowed since they are essential for health and well-being. People are urged to take care of their health in every area of life by the Holy Quran and the Hadith, which are important sources of information for Muslims regarding things pertaining to everyday life activities [30]. Additionally, God said in the Holy Quran, "Whoever saves a human life, it would be as if he saved all of mankind" (Quran, 5:32). Consequently, Islam supports vaccinations since they improve children's health and serve as a preventive tool against sickness. Furthermore, Islamic medical ethics uphold the principle that "necessity permits what is otherwise prohibited," emphasizing the importance of prioritizing health and well-being in cases of need [30].

Knowledge and education level of Saudi parents toward vaccinating preschool children

Parental knowledge, attitudes, and beliefs have a significant impact on when and how children should begin receiving vaccinations on the recommended schedule. According to Alruwaili et al. (2018) [7], 89.6% of parents thought that vaccines had a beneficial effect on children's health, and 85.6% of parents thought that immunizations were extremely important in reducing the spread of infectious illnesses. Accordingly, a study by Alfahl and Alharbil (2017) [6] found that although there was acceptable knowledge and attitudes regarding childhood vaccination, there were still gaps in attitudes that were attributed to various factors, such as culture, belief in healthcare providers, and safety of vaccines. The parents' degree of education and awareness may have an impact on their adherence to the immunization schedule for their children. For example, a study [20] indicated that Saudi parents had an adequate understanding of child vaccinations and a positive attitude about them. Higher parental education levels have been linked to favorable parental attitudes on child vaccination, according to a number of research studies done in the Kingdom of Saudi Arabia [20,31-33]. Similar studies [3-5,5,7,34-36] corroborated this finding that better-educated Saudi parents had positive attitudes and good knowledge regarding immunizing their children. In the central Saudi Arabian city of Riyadh, 87.3% of parents adhered to the vaccination schedule, according to a study that looked at parents' attitudes toward the acceptability, accessibility, and availability of immunization services [37]. On the other hand, a different study by Alfahl and Alharbi (2017) [38] revealed little parental vaccination awareness. Most of these studies showed that parents were aware of the beneficial role vaccinations play in illness prevention, notwithstanding some disparities in the findings. There are gaps in the particular understanding of some vaccinations, even with the high immunization rate and appropriate information [20]. Studies should primarily concentrate on undereducated populations and rural locations. Compared to those in rural areas, urban residents are more informed and have more optimistic views [3]. Furthermore, studies show that parents with less education than a university degree are linked to a higher risk of vaccine delays compared to parents with a university degree [39]. Researchers discovered that maternal education significantly improved infant immunization adherence in a related study conducted in Nigeria [40]. Furthermore, research conducted in Egypt found that half of the participating women who had completed college administered vaccinations to their kids on a regular basis. This outcome demonstrated the critical necessity for in-depth research on this matter because those numbers are significant. According to Ramadan et al. (2016) [41], almost 50% of the participants' children did not receive their vaccinations on schedule.

Technological factors

Research has shown that reliable information sources, such as social media, the internet, and television, are essential for raising public knowledge about childhood vaccinations [33]. Social media and the Internet are effective channels in Saudi society for disseminating health-related information, particularly those on kid vaccinations. In terms of where people get information about vaccinations, doctors at primary healthcare facilities rank third at 36 percent, followed by family members at 41.1% and media outlets at 19.6% and 3.3%, respectively. Furthermore, consistent results from the study by Al-Zahrani (2013) [33] showed that periodicals, TV, and the Internet were substantially connected with parents' positive attitudes toward children's immunization as sources of knowledge. According to another survey, 17.4% of Saudi parents get their knowledge about kid vaccinations from social media, compared to 14% from books, 9% from friends, and 1.5% from television [38] reported that young parents cited books and social media as their go-to sources for knowledge about kid vaccinations, whereas older parents turned to television. It is obvious that technology is essential to the communication of knowledge. The validity of information is a crucial consideration, even with the significant role social media plays in the spread of vaccine-related information. For example, social media sites like Facebook, YouTube, Instagram, and Twitter have revolutionized the way that information is disseminated. In certain ways, social media may be helpful, but it also aids in the dissemination of misleading information. It is directed at parents, alerting them about the risks associated with vaccinations for kids. Even though vaccination is required in the Kingdom of Saudi Arabia, anti-vaccination social activists use social media to instruct people on how to extract vaccine ingredients using traditional herbal therapy. This method may be potent enough to draw the ingredients out of the injection site and counteract the intended effects of the vaccine [42,43]. Parents' attitudes and opinions have been greatly impacted by the widespread social media claims concerning failing immunization efforts in the Kingdom of Saudi Arabia.

Knowledge gaps among healthcare providers

Knowledge is essential for healthcare professionals to increase patient confidence and awareness. According to Saudi Arabian research, medical professionals are ignorant of the value of some vaccines, such as the seasonal influenza shot [44]. One possible contributing reason to children's immunization delays might be ignorance. Notably, parents greatly trust healthcare professionals to offer advice, making them important stakeholders in immunization programs. Health workers such as doctors and nurses should spread the word about how important it is to get immunizations on time. Their lack of understanding may be the root of ongoing doubts about the safety of specific vaccinations, particularly when they are unable to address queries and worries regarding the advantages and disadvantages of vaccinations.

The research was carried out in Qatar with the objective of investigating the attitudes and views of pediatric healthcare providers toward influenza vaccination. It exposed misunderstandings and a poor acceptance rate for the seasonal influenza vaccination. Low acceptance rates were shown to be caused by a lack of trust between parents and healthcare practitioners [45]. The quality of care that was provided was improved by the patients' and healthcare professionals' efficient communication. According to cross-sectional research done in Saudi Arabia, mothers' access to health education is hampered by the fact that foreign nurses who do not understand Arabic are a source of both language and cultural obstacles [46-48]. Pediatricians occasionally don't listen to the difficulties or issues women have when caring for their kids. Because of the time constraints brought on by the requirement to visit more patients, physicians might not be able to provide moms with the information they want.

Analysis of the outcome

Age of the Parents

**Figure 2:**
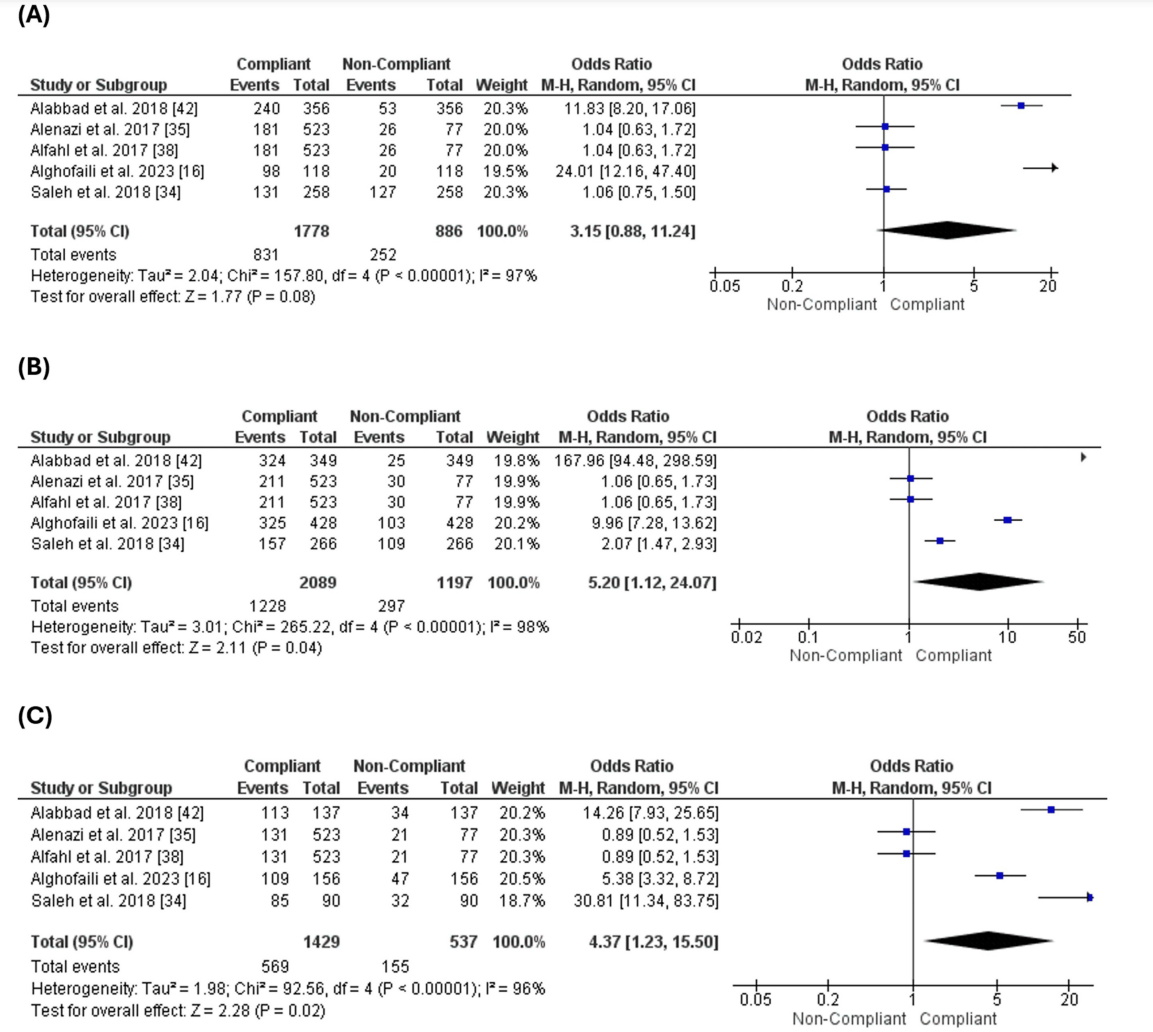
Age of the parents. Figure 2A: Compliance with vaccination for parents less than 30 years old. Figure 2B: Compliance with vaccination for parents between 30-40 years old. Figure 2C: Compliance with vaccination for parents more than 40 years old

Five studies [16,34,35,38,42] reported the age of the parents. Our analysis revealed the number of compliant parents was higher than that of non-compliant parents at ages less than 30. However, the difference between both cohorts was not significant (OR = 3.15 [0.88, 11.24], p = 0.08), and the pooled data were heterogeneous (p = 0.001, I2 = 97%) (Figure 2A).

On the other hand, the number of compliant parents was significantly higher than the number of non-compliant parents at the ages of 30-40 (Figure 2B) or more than 40 years old (Figure 2C) (OR = 5.20 [1.12, 24.07], p = 0.04), (OR = 4.37 [1.23, 15.50], p = 0.02), respectively.

**Figure 3:**
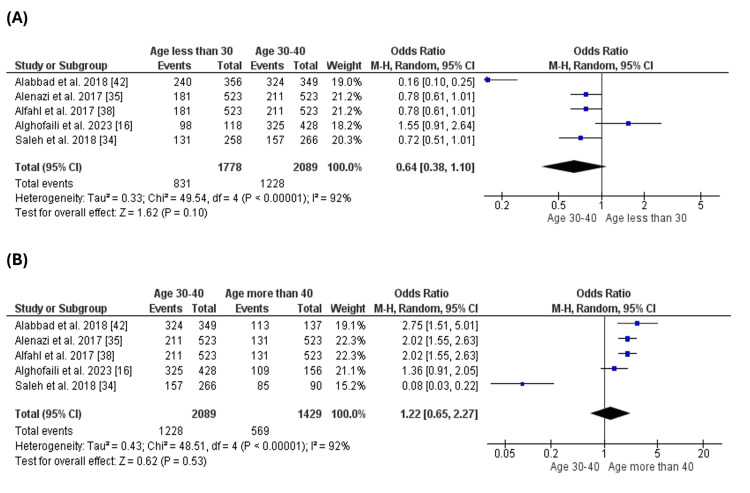
Comparison of compliance across different age groups. Figure 3A: Comparison of compliance between age less than 30 and age 30-40. Figure 3B: Comparison of compliance between ages 30 and 40 and ages more than 40

Comparing the number of complaints of parents of different ages, we found that there was a similarity between different ages, such as the ages less than 30, the ages between 30-40, or more than 40 years old (p-value = 0.1 and 0.5) (Figure 3). This analysis revealed that the parents' age does not affect their compliance with their children's vaccination.

Education of the Parents

**Figure 4:**
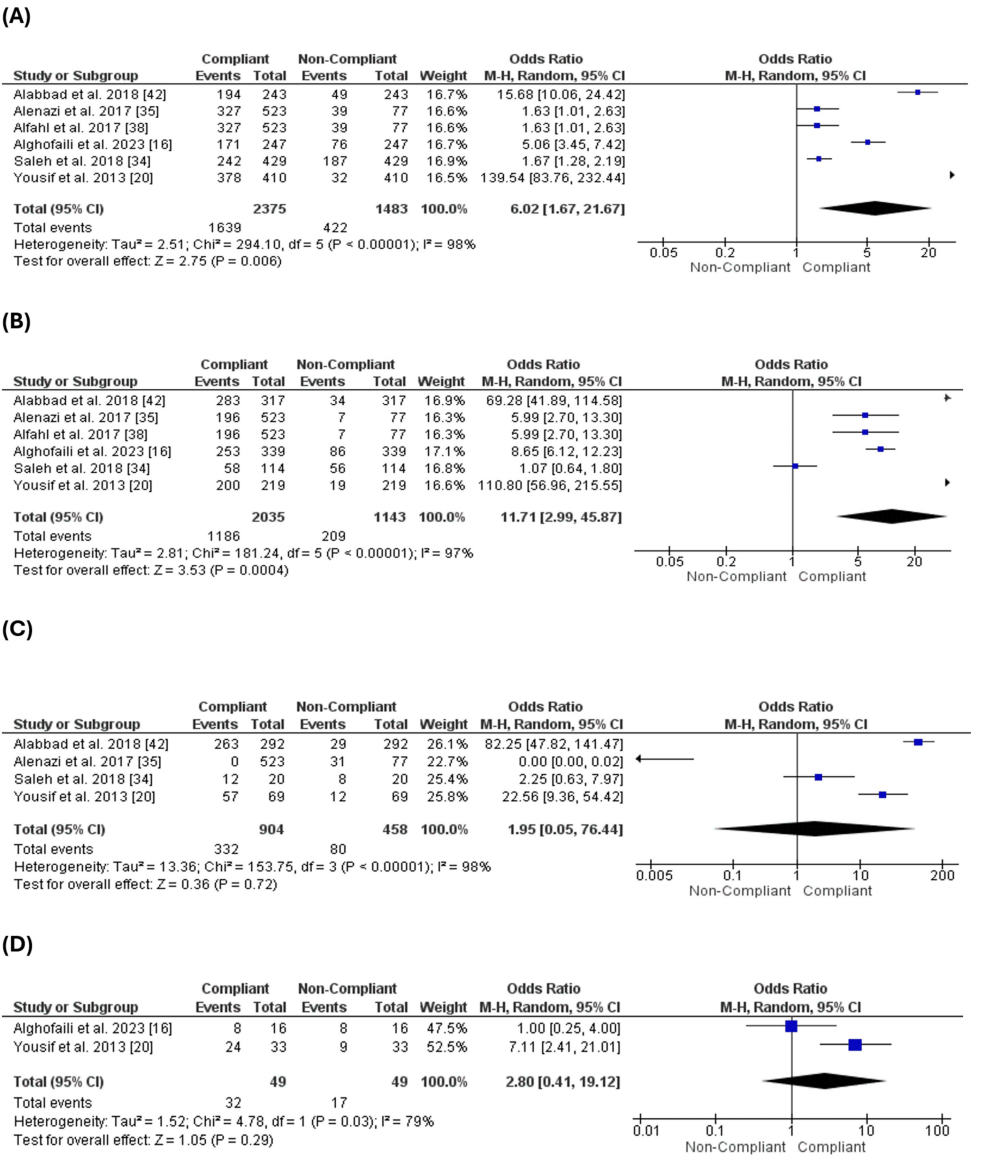
Education of the parents. Figure 4A: Compliance with vaccination for parents with university education. Figure 4B: Compliance with vaccination for parents with secondary school education. Figure 4C: Compliance with vaccination for parents with primary school education. Figure 4D: Compliance with vaccination for parents with no education

Six studies [16,20,34,35,38,42] reported the levels of education of the parents and evaluated it as a factor that may impact the parent's compliance with the vaccination of their children. The number of complaint parents was significantly higher than the number of non-compliant parents with different levels of education, such as university (OR= 6.02 [1.67, 21.67], (p=0.006) (Figure 4A), secondary school (OR= 11.71 [2.99, 45.87], (p=0.0004) (Figure 4B). However, there was no significant difference between the number of complaints and non-compliant parents who went to primary school (OR= 1.95 [0.05, 76.44], (p=0.72) (Figure 4C) or who did not get any education (OR= 2.80 [0.41, 19.12], (p=0.29) (Figure 4D).

**Figure 5:**
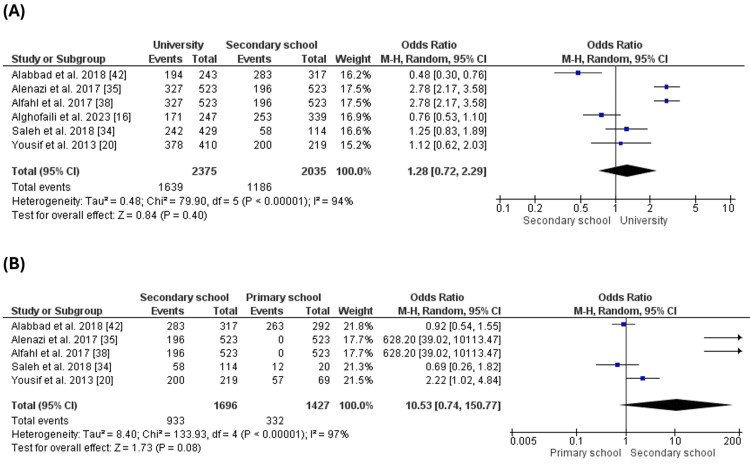
Comparison of compliance across different levels of education. Figure 5A: Comparison of compliance between university and secondary school. Figure 5B: Comparison of compliance between secondary school and primary school.

Comparing the different levels of education, we found that the number of complaint parents who went to colleagues was higher than the number of parents who went to secondary school (OR = 1.28 [0.72, 2.29], (p = 0.4), which was higher than the number of parents who went to primary school only (OR = 10.53 [0.74, 150.77], (p = 0.08) but there was no significant difference between them. At the same time, the number of compliant parents was the lowest with the parents who did not go to school or get any education (Figure 5). The analysis revealed that the level of education may affect parents' compliance with vaccinating their children.

Occupation of the Mothers

**Figure 6:**
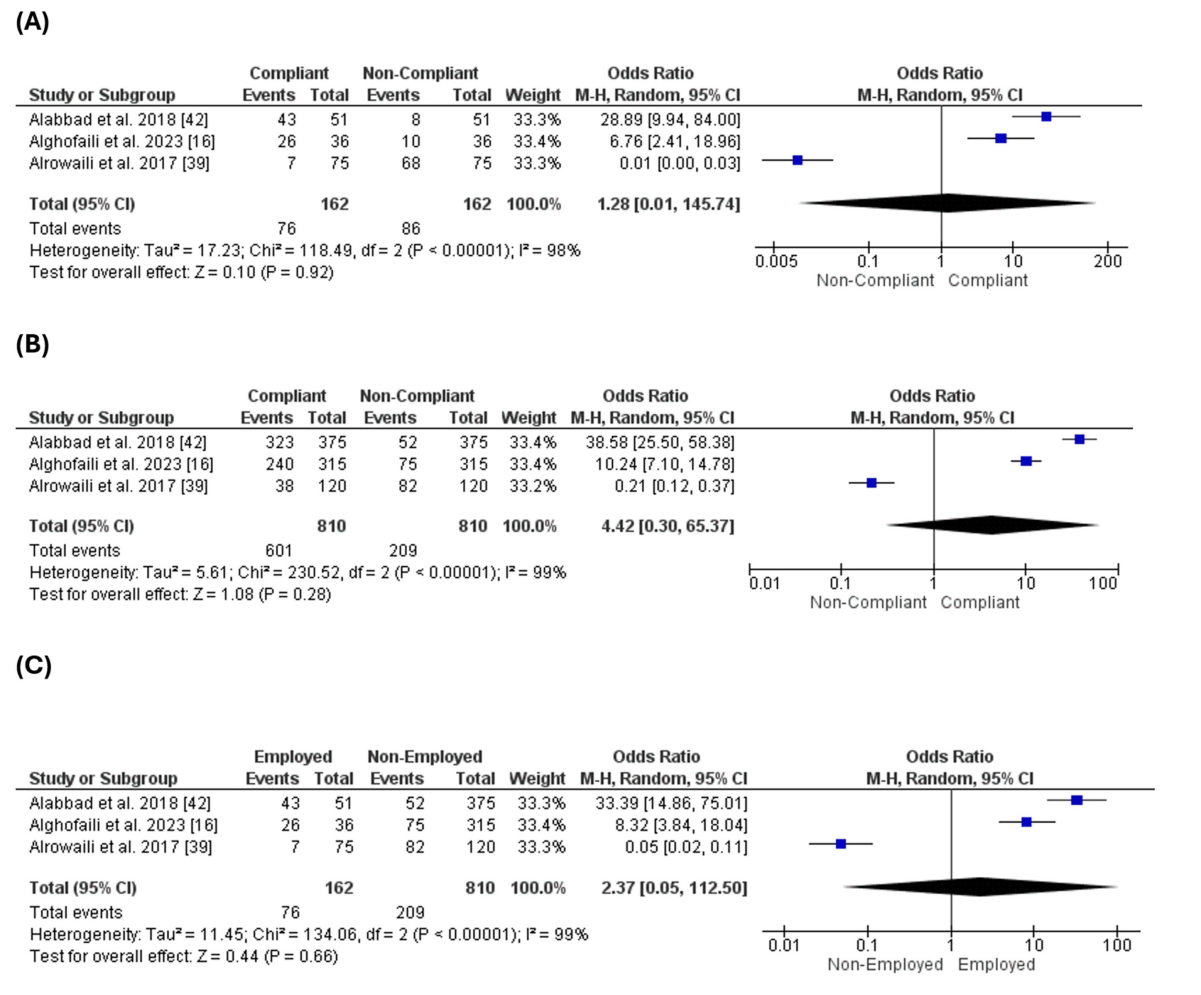
Occupation of the mothers. Figure 6A: Comparison of compliance with vaccination between employed mothers. Figure 6B: Comparison of compliance with vaccination between non-employed mothers. Figure 6C: Comparison of compliant mothers between employed and non-employed cohorts

Three studies [16,39,42] reported the occupations of mothers. There were no substantial variations in the number of compliant and non-compliant mothers in the group of employed mothers (p = 0.92) (Figure 6A). Additionally, there were no substantial variations in the number of compliant and non-compliant mothers in the group of non-employed mothers (p = 0.2) (Figure 6B). Also, there were no significant variations in the number of compliant mothers between the employed and non-employed cohorts (p = 0.66) (Figure 6C).

Occupation of the Fathers

**Figure 7:**
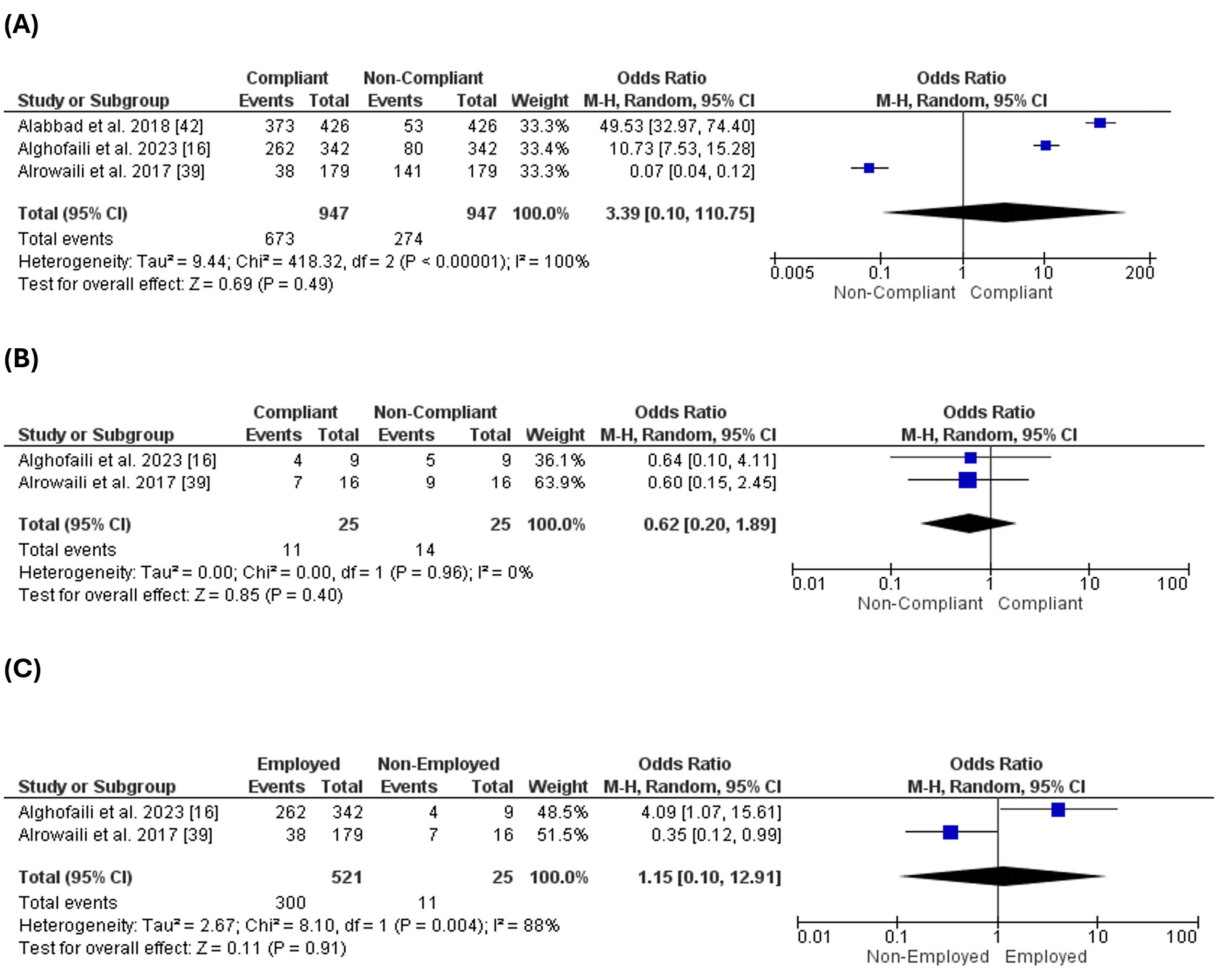
Occupation of the fathers. Figure 7A: Comparison of compliance with vaccination for employed fathers. Figure 7B: Comparison of compliance with vaccination for non-employed fathers. Figure 7C: Comparison of compliance with vaccination between employed and non-employed fathers

Three studies [16,39,42] reported the occupations of fathers. There were no substantial variations in the number of the complaint fathers and non-complaint fathers in the group of employed fathers only (OR = 0.24 [-0.46, 0.93], (p = 0.51) (Figure 7A). Additionally, there were no substantial variations in the number of the complaint fathers and non-complaint fathers in the group of non-employed fathers only (OR = 0.62 [0.20, 1.89], (p = 0.4) (Figure 7B). Also, there were no significant variations in the number of complaint fathers between the employed and non-employed cohorts only (OR = 1.15 [0.10, 12.91], (p = 0.91) (Figure 7C).

The Number of Children

**Figure 8:**
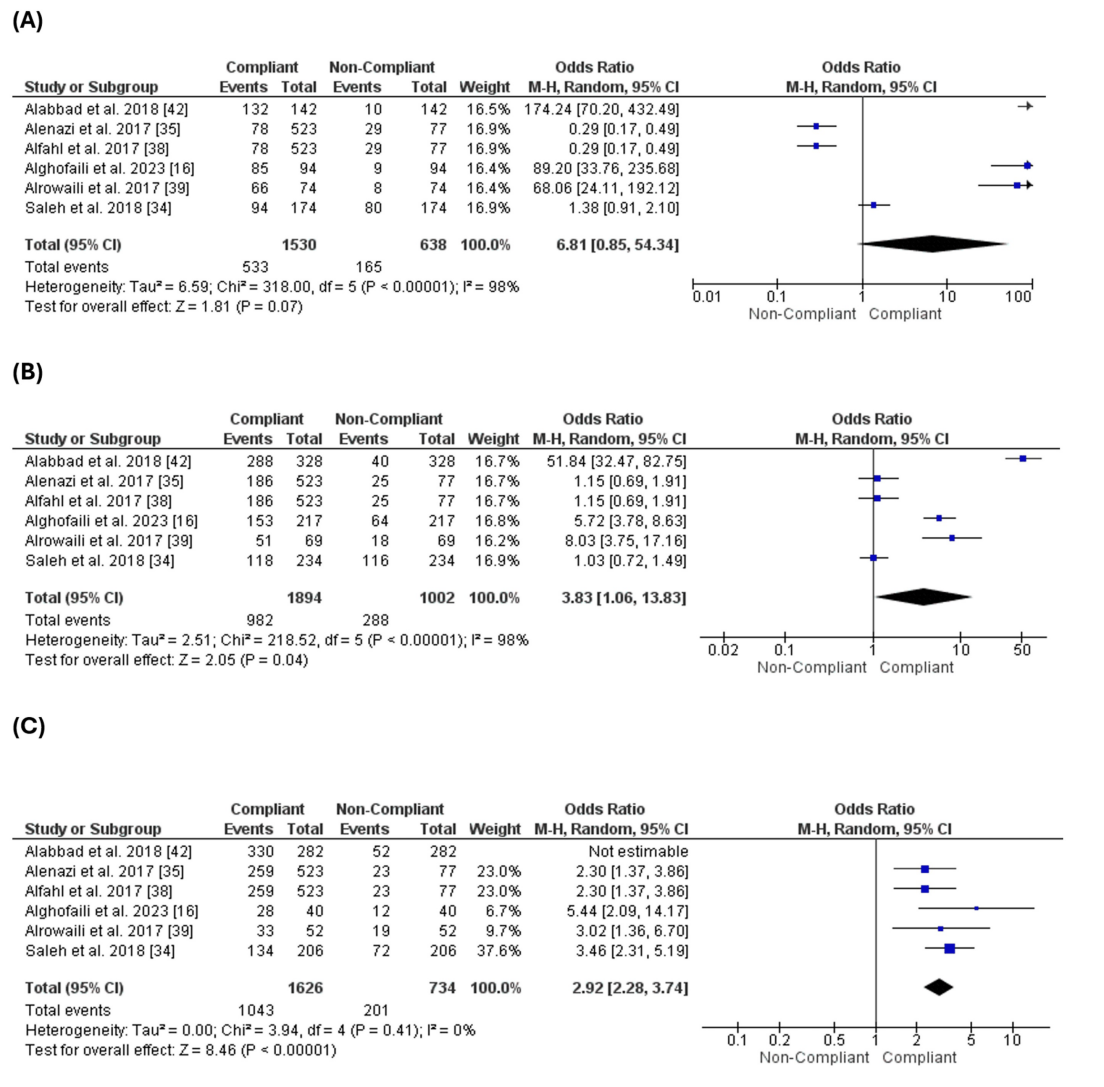
Comparison of compliance with vaccination according to the number of children. Figure 8A: Comparison of compliance with vaccination for parents with one child. Figure 8B: Comparison of compliance with vaccination for parents with 2-4 children. Figure 8C: Comparison of compliance with vaccination for parents with more than four children

**Figure 9:**
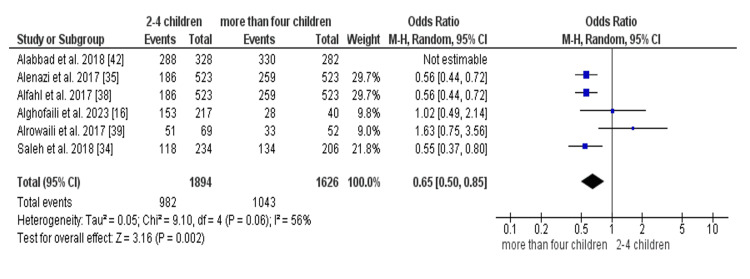
Comparison of compliance with vaccination between parents with more than four children and parents with 2-4 children

Six studies [16,34,35,38,39,42] reported this outcome. Comparing the parents who had one child, the number of complaint parents was similar to the number of non-compliant parents (OR = 6.81 [0.85, 54.34], (p = 0.07) (Figure 8A). In contrast, the group who had 2-4 children showed a higher number of complaint parents than the non-complaint (OR = 3.83 [1.06, 13.83], (p = 0.04) (Figure 8B). Additionally, the number of complaint parents who had more than four children was significantly higher than the number of non-compliant parents (OR = 2.92 [2.28, 3.74], (p = 0.0001) (Figure 8C). Also, the number of complaint parents who had more than four children was significantly higher than the number of complaint parents who had 2 to 4 children (OR = 0.65 [0.50, 0.85], (p = 0.002) (Figure 9).

Quality assessment

Table 2 displays the summary of the quality assessment of the included studies. Nearly all the studies were judged to be of medium quality, and only seven studies [8,16,20,28,32,33,39] were deemed to be of high quality. All the studies except Al-Shammari et al. 1991 [32] lost the quality points of the comparability domain. Regarding the selection domain, six studies [3,8,20,28,33,39] were awarded the full score, while the rest lost a quality point for not justifying the method of sample size calculation. All the studies except six studies [3-5,22,23,47] were awarded the full score in the outcome domain.

**Table 2:** Quality assessment of included studies assessed by the Newcastle-Ottawa scale

Study ID	Selection	Comparability	Outcome	Total score
Alsalmi et al. 2018 [3]	★★★★★	☆☆	★★☆	7
Alshammari et al. 2018 [4]	★☆★★★	☆☆	★★☆	6
Alshammari et al. 2018 [5]	★☆★★★	☆☆	★★☆	6
Alyami et al. 2017 [6]	★☆★★★	☆☆	★★★	7
Alruwaili et al. 2018 [7]	★☆★★★	☆☆	★★★	7
Alouf et al. 2019 [8]	★★★★★	☆☆	★★★	8
Al-Saeed et al. 2018 [12]	★☆★★★	☆☆	★★★	7
Hasanain et al. 2002 [15]	★☆★★★	☆☆	★★★	7
Alghofaili et al. 2023 [16]	★★★★★	☆☆	★★★	8
Al Tabbal et al. 2017 [17]	★☆★★★	☆☆	★★★	7
Yousif et al. 2013 [20]	★★★★★	☆☆	★★★	8
Abahussain et al. 2005 [22]	★☆★★★	☆☆	★★☆	6
Al-Bannay et al. 2017 [23]	★☆★★★	☆☆	★★☆	6
Deeb et al. 1997 [26]	★☆★★★	☆☆	★★★	7
Sinuraya et al. 2022 [28]	★★★★★	☆☆	★★★	8
Al-Shammari et al. 1991 [32]	★☆★★★	★☆	★★★	8
Al-Zahrani et al. 2013 [33]	★★★★★	☆☆	★★★	8
Saleh et al. 2018 [34]	★☆★★★	☆☆	★★★	7
Alenazi et al. 2017 [35]	★☆★★★	☆☆	★★★	7
Al Amri et al. 2018 [36]	★☆★★★	☆☆	★★★	7
Habib et al. 2018 [37]	★☆★★★	☆☆	★★★	7
Alfahl et al. 2017 [38]	★☆★★★	☆☆	★★★	7
Alrowaili et al. 2017 [39]	★★★★★	☆☆	★★★	8
Balogun et al. 2017 [40]	★☆★★★	☆☆	★★★	7
Ramadan et al. 2016 [41]	★☆★★★	☆☆	★★★	7
Alabbad et al. 2018 [42]	★☆★★★	☆☆	★★★	7
Alhammadi et al. 2015 [45]	★☆★★★	☆☆	★★★	7
Khattab et al. 2000 [46]	★☆★★★	☆☆	★★★	7
Halligan et al. 2006 [47]	★☆★★★	☆☆	★★☆	6
Sagor et al. 2018 [48]	★☆★★★	☆☆	★★★	7

Study ID	Selection	Comparability	Outcome	Total score
Alsalmi et al. 2018 [3]	★★★★★	☆☆	★★☆	7
Alshammari et al. 2018 [4]	★☆★★★	☆☆	★★☆	6
Alshammari et al. 2018 [5]	★☆★★★	☆☆	★★☆	6
Alyami et al. 2017 [6]	★☆★★★	☆☆	★★★	7
Alruwaili et al. 2018 [7]	★☆★★★	☆☆	★★★	7
Alouf et al. 2019 [8]	★★★★★	☆☆	★★★	8
Al-Saeed et al. 2018 [12]	★☆★★★	☆☆	★★★	7
Hasanain et al. 2002 [15]	★☆★★★	☆☆	★★★	7
Alghofaili et al. 2023 [16]	★★★★★	☆☆	★★★	8
Al Tabbal et al. 2017 [17]	★☆★★★	☆☆	★★★	7
Yousif et al. 2013 [20]	★★★★★	☆☆	★★★	8
Abahussain et al. 2005 [22]	★☆★★★	☆☆	★★☆	6
Al-Bannay et al. 2017 [23]	★☆★★★	☆☆	★★☆	6
Deeb et al. 1997 [26]	★☆★★★	☆☆	★★★	7
Sinuraya et al. 2022 [28]	★★★★★	☆☆	★★★	8
Al-Shammari et al. 1991 [32]	★☆★★★	★☆	★★★	8
Al-Zahrani et al. 2013 [33]	★★★★★	☆☆	★★★	8
Saleh et al. 2018 [34]	★☆★★★	☆☆	★★★	7
Alenazi et al. 2017 [35]	★☆★★★	☆☆	★★★	7
Al Amri et al. 2018 [36]	★☆★★★	☆☆	★★★	7
Habib et al. 2018 [37]	★☆★★★	☆☆	★★★	7
Alfahl et al. 2017 [38]	★☆★★★	☆☆	★★★	7
Alrowaili et al. 2017 [39]	★★★★★	☆☆	★★★	8
Balogun et al. 2017 [40]	★☆★★★	☆☆	★★★	7
Ramadan et al. 2016 [41]	★☆★★★	☆☆	★★★	7
Alabbad et al. 2018 [42]	★☆★★★	☆☆	★★★	7
Alhammadi et al. 2015 [45]	★☆★★★	☆☆	★★★	7
Khattab et al. 2000 [46]	★☆★★★	☆☆	★★★	7
Halligan et al. 2006 [47]	★☆★★★	☆☆	★★☆	6
Sagor et al. 2018 [48]	★☆★★★	☆☆	★★★	7

Discussion

The numerous studies carried out in Saudi Arabia make it clear that the government has given regular childhood vaccinations the highest priority. Although vaccination rates are high, there are instances of children not receiving their recommended vaccinations on time, and some parents have doubts about the value of following regular immunization regimens [8]. According to research by Alruwaili et al. (2018) [7], 9% of parents would not urge their relatives to take their children to get vaccinated, which serves as evidence of parent vaccine hesitation. These problems indicate a lack of understanding of the significance of vaccination as well as some underlying resistance to the immunization effort. Some parents showed poor faith in the vaccination programs, according to the report. This might be because of social and cultural factors that politicians and medical experts have not addressed. According to Alruwaili et al. (2018) [7]. Parents generally understand and embrace the need for regular childhood vaccines, but, to achieve 100% compliance, parental awareness must be raised. Nevertheless, there is a gap in the research since it did not investigate the reasons behind certain parents' lack of enthusiasm for finishing the schedule and their lack of support for the program.

Regardless of the research year, a number of Saudi Arabian studies have found high levels of acceptability for regular childhood vaccination [7,10,16]. Furthermore, although research was conducted in both urban and rural areas, there was no comparison of the regional variations in socio-cultural determinants. For instance, regional differences exist in the ways that educational attainment, cultural influences, and spiritual inclinations affect local belief systems. There was a difference in the opinions of persons living in rural and urban areas, according to the assessment of knowledge and attitudes about child immunization. According to Alsalmi et al. (2018) [3], informed individuals in metropolitan areas often exhibit more optimistic attitudes than those in rural areas. Furthermore, although the majority of Saudi Arabia's population is Muslim, there are still antiquated health beliefs that might make it difficult to promptly follow a child's immunization schedule. Regretfully, most of the reviewed literature confirms that relatively few research studies have examined Saudi parents' perspectives on the cultural and religious justifications for delaying immunizations. Cultural overtones have often been labeled as "other" without a thorough analysis of how much they affect timely vaccination adherence.

Furthermore, research has concentrated on children's immunization records that are required before they enter school. The unexpected impacts that a kid may experience from infancy till school age were not considered in these investigations. Given that just the required vaccines are required before reaching school age, this fact raises the question of whether the requirement might be interpreted incorrectly. While following the law accomplishes a significant coverage milestone, it ignores a crucial component of the benefits of vaccination throughout an individual's lifetime. Based on the knowledge that cultural and societal attitudes should be fundamental in establishing herd vaccination at any age range, culturally congruent treatment becomes necessary on these boundaries. For example, a study's findings have shown that people had low rates of adherence to the voluntary seasonal influenza vaccination program [47]. According to this study, 63.3% of the participants had never had an influenza vaccination, endangering the safety of the population.

In terms of the analysis, our research showed a substantial correlation between the parents' age and their adherence to their children's vaccination schedule. Parents older than forty or older than 30 had a higher likelihood of being obedient than parents younger than 30. According to earlier studies [3-5,10,16,18,34,38], older parents are more likely to be aware of the advantages of vaccination and to have the resources to access vaccination services. This conclusion is consistent with those studies. This might be caused by a variety of things, such as the fact that older parents have had more vaccination experience, are more aware of the advantages of vaccinations, and are more concerned about illnesses that can be prevented by vaccination. Moreover, parents who are older may have greater incomes and educational attainments, which have been connected to higher vaccination rates in the past [48].

In addition, the level of education of the parents was also significantly associated with their compliance with vaccinating their children. Parents who went to university or secondary school were more likely to be compliant than parents who went to primary school or who did not get any education. Our results are consistent with previous research, which has shown that education is a strong predictor of health behaviors [20,31,33].

The number of children a parent had was also significantly associated with their compliance with vaccinating their children. Parents who had more than four children were more likely to be compliant than parents who had one child or 2-4 children. 

Regarding the occupation of the mothers and fathers, we found that it was not significantly associated with their compliance with vaccinating their children. However, the findings of the present study suggest that the occupation of the parents may not be a major factor in determining compliance with vaccination [8].

Implications for nursing

Implications for Education

This review serves as an example of how culture affects vaccination program adherence and as a foundation for developing and putting into practice vaccine adherence-promoting methods. It is clear from the Sunrise Enabler-Model interpretative analysis that it is possible to address the intention to follow the immunization schedule. Therefore, to close the gaps resulting from problems with vaccination perception and safety concerns, educational initiatives are required. The answers might be achieved by launching parent awareness programs that emphasize the value of vaccinations and by educating medical professionals so they can inform parents with confidence about the necessity of vaccinating their children in accordance with the advised schedule. Additionally, the instruction ought to be compliant with the health-promoting legislation that is now in effect and is based on Saudi Arabia's Vision 2030. Health practitioners should get training to supplement planned childhood vaccination knowledge based on the Saudi Vision 2030 regulations. To give students a foundational understanding of culturally appropriate nursing care, a transcultural care education curriculum ought to be incorporated into nursing curricula.

To improve child vaccine adherence rates, the intervention's tactics should also consider the components of effective communication and creating awareness campaigns. Reducing vaccination-related anxieties and addressing vaccine hesitancy issues require accurate information from the appropriate source. Reluctance to receive vaccinations is still a global problem that requires more attention. Participants' levels of trust in doctors and the KSA's Ministry of Health were found to be high. These results indicate that people's decisions on kid vaccinations are heavily influenced by information. Therefore, the problem of delays may be resolved if the Ministry of Health (MOH) and the healthcare providers work together to raise awareness.

It is recommended that nurses and other healthcare professionals actively engage in awareness campaigns, utilizing diverse technological platforms to spread genuine messages and encourage adherence to immunization regimens. Smartphone applications that promote vaccination adherence can be utilized as instructional resources and reminders to improve vaccine adherence [49]. The government should consciously allocate funds to immunize the populace against childhood vaccinations.

Implications for Policy

Because this study offers a broad review of the cultural influences globally, as well as in the Arabic Gulf area particularly, health policy professionals will be better equipped to create targeted interventions that encourage vaccination schedule adherence. It makes sense that children who are required to be vaccinated before being admitted to school have benefited from high vaccination rates across the country. Nonetheless, the legislation hasn't addressed localized anti-vaccine sentiment. One example of coverage advantages gained as a result of legislative initiatives is Saudi Arabia's need for preschool vaccinations [50].

Mandatory vaccination recommendations, however, do not deal with the problem of immunization delay. Thus, the first step in addressing vaccination delays and public doubts about the need and safety of certain vaccinations would be to comprehend cultural and social variables. For example, requiring Saudi Arabian children to obtain a vaccination certificate prior to starting school has only increased vaccination coverage from roughly 96% to 98% [(51,52]. However, can the government view routine vaccine adherence as a problem that has been solved and no longer calls for coercive approaches? This is a relevant subject that, to be effectively answered, requires a research-based strategy that delves further into the question's cultural components. The WHO (2019) [51,52] reports that vaccine coverage is now at a decent level. However, it is important to remember that herd immunization is only possible in situations when vaccinations are administered on time. Health professionals must examine the cultural aspects that different stakeholders should address in order to promote improved health-seeking behaviors since the present global health delivery system promotes the provision of healthcare that is culturally congruent [53]. Thus, addressing the cultural dimensions, which are absent from most studies, may help resolve safety concerns and guarantee immunization schedules on time, not just in Saudi Arabia but in other nations with comparable sociocultural problems.

Implications for Research

As has been previously said, rather than examining the cultural component of vaccine reluctance, most of the research included in this review concentrated on one aspect: parental knowledge. Furthermore, most of the research carried out in Saudi Arabia only included a portion of the population, not the entire population, so it was impossible to provide a meaningful statistical representation of this issue. Thus, further research is required to examine health system accessibility in Saudi Arabia's rural areas to address the issue of children's vaccinations being delayed. Furthermore, this study emphasizes the need for more research to examine the socio-cultural elements pertaining to social media, religion, and healthcare access that may be influencing the occurrence of this issue.

Limitations of the study

Considering the present review, some limitations may affect the generalization of the findings. Despite this paper being comprehensive by virtue of reviewing previous studies that have investigated the phenomenon of vaccine hesitancy, issues of completeness and biases arise. First, the completeness of the present review is only limited to the context of the accessed articles in specific countries. As a result, the biases and factors overlooked by previous researchers are likely to be replicated in the present study, and the results cannot be generalized worldwide because they focus only on Saudi culture and countries that have similar traditions. Further, most of these studies relied on small study samples that could limit the generalization of the findings to the vast Kingdom of Saudi Arabia. Bias would also exist because this paper does not cover different cultures’ perspectives. Besides, this review may have missed some relevant articles by virtue of lacking access, or they have not been published yet. The second limitation of this review is the focus on the general determinants and not specifically on the cultural issues that cause parents to delay their children’s vaccination; thus, the findings can only be described as tentative, and hence there is a need for more investigative qualitative studies. Thirdly, there is a possibility of respondent bias in the search, the identification, and the thematic analysis of the articles, which may affect the reliability of the study. This is because the entire review was undertaken by one reviewer; hence, it is devoid of objective analysis by other researchers. However, it is worth underscoring that the limitations may not interfere with the validity of the research.

## Conclusions

Parents in Saudi Arabia often delay vaccinations scheduled for preschool-aged children due to various reasons, primarily rooted in cultural factors rather than religious beliefs. Saudi parents' opinions and attitudes toward delayed vaccination are shaped by these cultural influences. For instance, in close-knit families, women may defer to their husbands or male guardians as the primary decision-makers, believing they should always be consulted. Additional factors include the influence of social media, which can sway vaccination decisions. Despite high vaccine adherence rates, the findings of this study highlight the need for targeted measures to address these cultural drivers. Furthermore, comprehensive qualitative research is essential to explore the deeper traditional aspects of Saudi society that influence vaccination practices.
